# Divergent Skull Morphology Supports Two Trophic Specializations in Otters (Lutrinae)

**DOI:** 10.1371/journal.pone.0143236

**Published:** 2015-12-09

**Authors:** Lori L. Timm-Davis, Thomas J. DeWitt, Christopher D. Marshall

**Affiliations:** 1 Department of Wildlife & Fisheries Sciences, Texas A&M University, College Station, TX, 77843, United States of America; 2 Department of Marine Biology, Texas A&M University, 200 Seawolf Parkway, Galveston, TX, 77553, United States of America; Max Planck Institute for Evolutionary Anthropology, GERMANY

## Abstract

Variation in terrestrial mammalian skull morphology is known to constrain feeding performance, which in turn influences dietary habits and ultimately fitness. Among mustelids, otters have evolved two feeding specializations: underwater raptorial capture of prey (mouth-oriented) and capture of prey by hand (hand-oriented), both of which have likely associations with morphology and bite performance. However, feeding biomechanics and performance data for otters are sparse. The first goal of this study was to investigate the relationships between feeding morphology and bite performance among two mouth-oriented piscivores (*Pteronura brasiliensis* and *Lontra canadensis*) and two hand-oriented invertebrate specialists (*Enhydra lutris* and *Aonyx cinerea*). Since other vertebrate taxa that are mouth-oriented piscivores tend to possess longer skulls and mandibles, with jaws designed for increased velocity at the expense of biting capability, we hypothesized that mouth-oriented otters would also possess long, narrow skulls indicative of high velocity jaws. Conversely, hand-oriented otters were expected to possess short, blunt skulls with adaptations to increase bite force and crushing capability. Concomitant with these skull shapes we hypothesized that sea otters would possess a greater mandibular bluntness index, providing for a greater mechanical advantage compared to other otter species investigated. A second goal was to examine morphological variation at a finer scale by assessing variation in cranial morphology among three sea otter subspecies. Since diet varies among these subspecies, and their populations are isolated, we hypothesized that the magnitude of mandibular bluntness and concomitant mechanical advantage, as well as occlusal surface area would also vary within species according to their primary food source (fish versus hard invertebrates). Functional expectations were met for comparisons among and within species. Among species the phylogeny suggests a deeply rooted transition to alternative foraging types. Yet within foraging types alternative species were also strongly variable, suggesting either selective differences in the extent or nature of realized foraging mode, or an accumulation of non-adaptive changes during the long independent evolutionary history. At the finest scale, variation among subspecies indicates that trophic adaptation occurred rapidly, making it interesting that we happened to find both deeply and shallowly-rooted transformations associated with diet type in otter species and subspecies.

## Introduction

Carnivora is one of the most ecologically diverse mammalian orders [1–3] with a fossil record that extends back 44 million years [3, 4]. Variation in skull morphology has been shown to constrain feeding performance [1, 3, 5–9], which in turn influences dietary habits, survival, and ultimately fitness [10–11]. Mustelidae (weasels, otters, badgers and skunks) is one of the most ecologically diverse families in the order Carnivora and provides interesting opportunities to explore the morphological and biomechanical diversity of feeding [6]. Although skull morphology and dietary adaptations have been examined to some extent in terrestrial mustelids [6,12–15] few data exist for aquatic mustelids such as otters (Lutrinae).

Morphological and behavioral diversity among otters is reflected in their diet and foraging behaviors [2–8, 16–18]. For example, in North American river otters (*Lontra canadensis*), the jaw muscles are hypertrophied compared to terrestrial carnivores, which is thought to allow the rapid jaw motion necessary for catching elusive fish with their mouths underwater [6]. This is reflected in their cranial morphology; river otters possess broad mastoid processes where the enlarged digastric muscles originate [6]. River otters also possess sharp carnassials necessary for piercing and shearing fish [14]. In contrast, sea otters (*Enhydra lutris*) possess short, blunt skulls with bunodont dentition, used for crushing hard, benthic prey [10, 19]. Despite numerous studies regarding durophagy and trophic ecology in otters, there are few data on the functional morphology of feeding in otters [20]. Otters are often categorized into two trophic specializations: mouth-oriented piscivory or hand-oriented invertebrate specialists [21–22]. North American river otters, neotropical river otters (*Lontra longicaudis*), giant river otters (*Pteronura brasiliensis*), smooth coated otters (*Lutrogale perspicillata*), European river otters (*Lutra lutra*), and hairy-nosed otters (*Lutra sumatrana*) are considered mouth-oriented predators [23]. Mouth oriented feeding is considered the basal feeding mode [21, 24], and species that use this mode are primarily piscivores [22]. Based on diet alone, it is likely that spotted-necked otters (*Hydrictis maculicollis*) are also mouth-oriented predators, since they feed primarily on fish, frogs, and amphibians [25]. In contrast, sea otters (*Enhydra lutris*), Asian small-clawed otters (*Aonyx cinerea*), and African clawless otters (*Aonyx capensis*) primarily feed on invertebrate prey [22] and are considered to be hand-oriented predators [23, 26–27]. Although Asian small-clawed otters primarily feed on invertebrates, they do incorporate fish into their diets [26], as do some populations of sea otters [28]. We predicted that the underlying skull morphology and biomechanics will correlate with the feeding performance of these two feeding specializations (i.e., mouth-oriented piscivory and hand-oriented invertivory).

Our first objective was to compare skull morphology among two mouth-oriented, piscivore specialists (North American river otters and giant river otters) and two hand-oriented invertebrate specialists (Asian small-clawed otters and sea otters) to investigate the basis for their mechanical diversity [6] and dietary specializations [29]. We hypothesized that mouth-oriented otters possess long, narrow skulls indicative of high velocity jaws in contrast to hand-oriented otters which we hypothesized possess short, blunt skulls and a greater occlusal surface area for crushing capability. Additionally we predicted that sea otters possess the greatest mandibular bluntness index [30] compared to all otter species investigated and that sea otters would possess a greater mechanical advantage than other otter species investigated.

Our second objective was to investigate variation in skull morphology among three sea otter subspecies (northern sea otters, *Enhydra lutris kenyoni*; southern sea otters, *Enhydra lutris nereis*; and Russian sea otters, *Enhydra lutris lutris*). These subspecies exist in geographically isolated populations [29]. Although sea otters are typically thought of as durophagous predators that feed on hard, benthic prey, northern sea otters distributed within the Aleutian Islands (southwestern stock) and Russian sea otters do incorporate fish into their diets [28]. Although certain stocks in Alaska incorporate fish into their diets, in general, Alaskan sea otters prey on benthic invertebrates. In contrast, Russian sea otters incorporate a greater proportion of fish into their diet compared to northern and southern sea otters. This is in contrast to southern sea otters, which primarily prey on hard benthic invertebrates such as clams, abalone, and sea urchins [31–33]. Therefore, considering dietary differences and geographic isolation, how does cranial morphology differ among these subspecies? We hypothesized that the magnitude of mandibular bluntness and mechanical advantage, as well as occlusal surface areas, would vary among subspecies concomitant with their prey sources (fish versus hard invertebrates). Southern sea otters should possess shorter and blunter skull morphologies and shapes with a greater mechanical advantage and greater occlusal surface area, relative to the other otter subspecies, especially Russian sea otters, which tend to have a greater proportion of fish in their diets.

## Materials and Methods

### Samples

One hundred fifty skulls from four otter species (2 mouth-oriented and 2 hand-oriented) were examined. Species were chosen to include the wide breadth of feeding adaptations in otters. North American river otters (n = 43) and giant river otters (n = 17) were chosen to represent mouth-oriented piscivore specialists. Hand-oriented otters were represented by Asian small-clawed otters (n = 23), and all 3 subspecies of sea otters (northern, n = 40; Russian, n = 8; southern, n = 20). Specimens were obtained through loans courtesy of the Burke Museum of Natural History (Seattle, WA, USA), National Museum of Natural History (Washington, D.C., USA), and the American Museum of Natural History (New York, NY, USA). Accession numbers for all specimens appear in the included data file (S1 Dataset).

**Fig 1:**
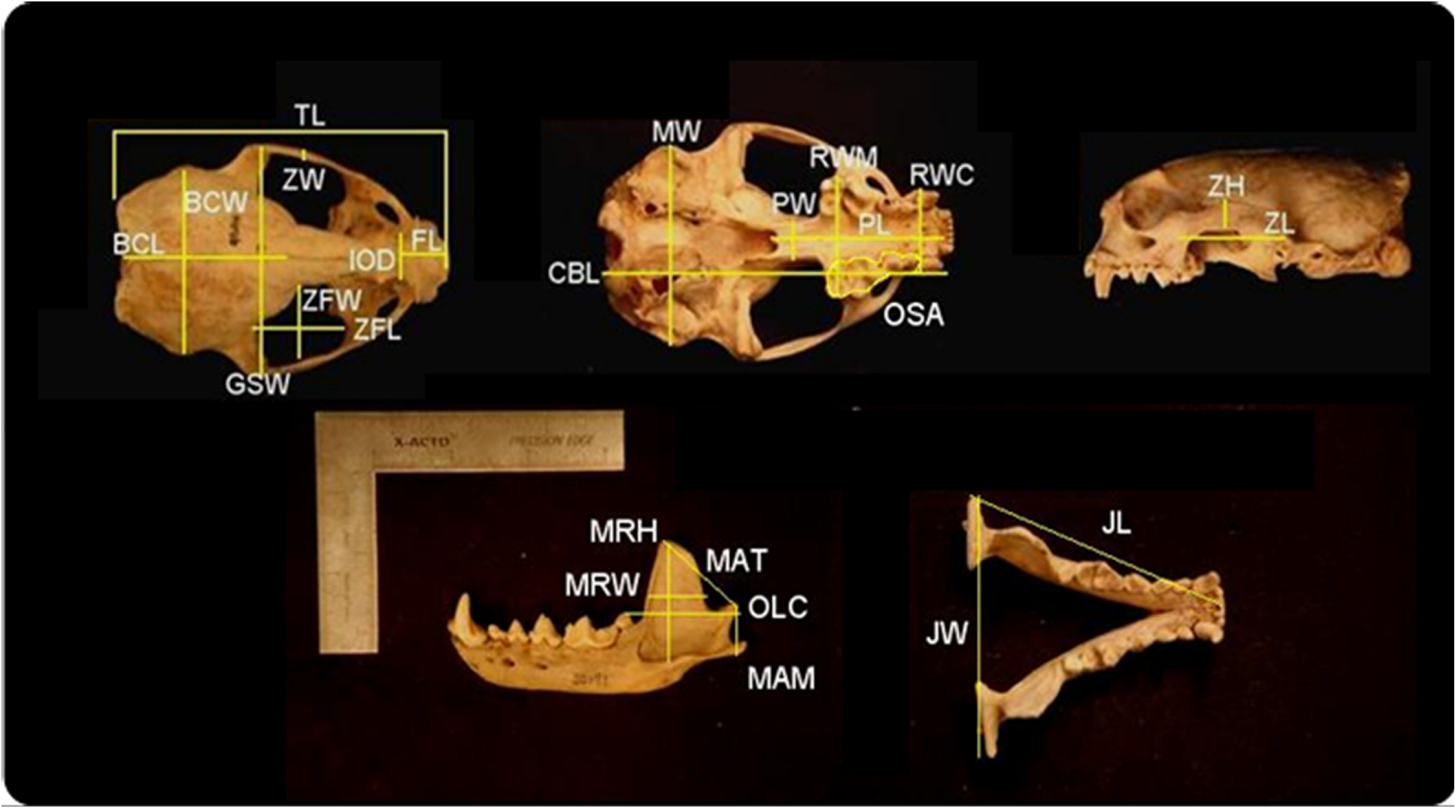
Morphometric measurements. TL, total length of skull; BCL, brain case length; BCW, brain case width; ZW, zygoma width; FL, face length; IOD, inter-ocular distance; ZFW, zygomatic fossa width, ZFL, zygomatic fossa width, GSW, greatest skull (squamosal) width; MW, width at mastoids; PW, palate width; RWM, rostral width at molars; PL, palate length; RWC, rostral width at canines; PL, palate length; CBL, condylobasal length; OSA, occlusal surface area of premolar and molar teeth; ZH, zygoma height; ZL, zygoma length; MRW, mandibular ramus width; MRH, mandibular ramus height; MAT, moment arm of the temporalis; OLC, out lever at the condyle; MAM, moment arm of masseter; JW, jaw width; JL, jaw length. Trait definitions are given in Table 1.

Twenty-five standard skull measurements (Fig 1; Table 1), following references [5], and [34–36], were collected using digital calipers (Mitutoyo, Aurora, IL) (0.001 mm). Occlusal surface area of premolar and molar teeth together was measured using scaled digital images in Image J (NIH, Bethesda, MD) and converted to its square root, which linearized the measure for inclusion with other single dimension linear distances in the analysis(e.g. [37]). Mandibular bluntness index (**MBI**) was calculated using scaled digital photographs and ImageJ following Werth [30]. The MBI calculates a ratio of jaw width to length, which is more reliable for kinematic inference than skull width to length ratios [30]. The ratio of the distance between the two condylar edges (**JW**), and the distance from the anterior tip of the mandibular symphysis to the posterior edge of the mandibular condyle (**JL**) were measured.

**Table 1:** Definitions of morphometric variables.

**Total length (TL)**	Maximum length of skull from tip of rostrum to the nuchal crest	**Greatest squamosal width (GSW)**	Maximum width of zygomatic arches dorsal to glenoid fossa
**Braincase length (BCL)**	Apex of nuchal crest to postorbital constriction	**Zygoma width (ZW)**	Maximum width or thickness of zygomatic arch at jugal-squamosal suture
**Braincase width (BCW)**	Greatest width across braincase posterior to zygomatic arches and dorsal to tympanic bullae	**Zygoma length (ZL)**	Maximum length including squamosal and jugal
**Condylobasal length (CBL)**	Distance from posterior projections of the occipital condyles to anterior edge of premaxillary bones	**Zygoma height (ZH)**	Maximum height of zygomatic arch at jugal-squamosal sture
**Face length (FL)**	Fronto-nasal suture to anterior most tip of premaxilla	**Zygomatic fossa width (ZFW)**	Maximum width of zygomatic fossa from directly posterior to molar fossa near frontal bone
**Interorbital distance (IOD)**	Least distance across orbits, anterior to post-orbital process	**Zygomatic fossa length (ZFL)**	Maximum length of zygomatic fossa from posterior/lateral of molar fossa to anterior of glenoid fossa
**Palatal length (PL)**	Alveolus of first incisors to anteriormost point on posterior edge of palate	**Jaw width (JW)**	Distance from most posterior part of condyle to posterior part of condyle
**Palatal width (PW)**	Width across palate posterior to last upper molars	**Jaw length (JL)**	Distance from anterior tip of mandibular symphysis to posterior edge of mandibular condyle
**Rostral width at canine (RWC)**	Maximum width of rostrum at canines; including canines	**Mandibular ramus height (MRH)**	Maximum height of ramus from apex of coronoid process to deepest point of masseteric fossa
**Rostral width at molars (RMW)**	Maximum rostral width at last upper molars; including molars	**Mandibular ramus width (MRW)**	Maximum width of ramus from interior condyle process to edge of coronoid process
**Mastoid width (MW)**	Width from mastoid to mastoid	**Moment arm of masseter (MAM)**	Distance from ventral border of angular process to dorsal tip of condyle process
**Occlusal surface area (OSA)**	Surface of postcanine tooth row	**Moment arm of temporalis (MAT)**	Distance from dorsal border of coronoid process to dorsal tip of condyle process
**Outlever at carnassial (OLC)**	Distance from condyle to posterior carnassial		

The mechanical advantages (**MA**) of the masseter and temporalis muscles were calculated for each specimen (following [4, 38]). The in-lever arm length (moment arm of masseter, **MAM**; moment arm of temporalis, **MAT**) was measured as the distance from the jaw joint to the insertion points of each muscle [4, 34–39]. The out-lever was measured as the distance from the jaw joint to the bite point (posterior carnassials) on the mandible [4]. More specifically, MAM was measured as the distance between the dorsal surface of the mandibular condyle to the ventral border of the angular process (Fig 1). Therefore, the mechanical advantage of the masseter (MA_masseter_) was measured by dividing the MAM by the distance from the posterior-most condyle process edge to the posterior of the lower carnassial (**OLC**) as follows:

MAmasseter=MAMOLC

MAT was measured as the distance between the dorsal surface of the mandibular condyle to the apical tip of the coronoid process (Fig 1). The mechanical advantage of the temporalis (MA_temporalis_) was measured by dividing the MAT by OLC as follows:

MAtemporalis=MATOLC

### Data Imputation and Transformations

Bilateral measures were taken on both sides of the skull but averaged for analysis. For a small minority of cases (0.6%), measures were only obtainable on one side of the skull and these were used alone as our measure for the variable. Twenty five specimens were missing one or a few measures due to skull damage or deterioration, producing a missing data frequency of just under 1%. Rather than exclude the 25 specimens missing a minority of data, we imputed values using maximum likelihood in JMP (SAS Inst., Cary, NC). Linear measurements were first transformed to the natural (base *e*) logarithm, hereafter “log”, and species identities were encoded as a design matrix using effects coding before imputation. Imputed values exert vanishingly small leverage in analysis because they are expected values given the overall (independent and dependent variable) data structure, yet they allow analysis with the informative majority of measurements of all 150 specimens [40].

### Size and Shape

To enable separate inference regarding size and shape, and statistical tests and control for allometry, skull form data were decomposed into size and shape. Definitions of size in traditional morphometric analyses are often problematic or contentious (see discussions by [41–42]). These issues are not a problem in geometric morphometrics because centroid size, effectively a measure of object area (in two dimensions) or volume (in three dimensions), is accepted as an unbiased measure of size [43–44]. In traditional morphometrics, body mass is relatively uncontroversial as a size measure but is often unavailable, for example, when working with museum preparations. For structures like skulls, a clear measure of size is object “volume”, taken to mean the space occupied by the structure, which is analogous to centroid size in geometric morphometrics. Object volume should be highly related to object mass. A cubic centimeter of volume should translate closely to mass in grams, as the average density of biological objects is near that of water (1 cm^3^ tissue = 1 g mass). An estimate of object volume is the product of major linear dimensions like length, width and depth measures. Since volumes are multidimensional, however (e.g., cm^3^), they must be linearized to be used in analyses with linear measures. The linear measure of a multidimensional volume is the geometric mean of the major dimensions of an object—the *k* ^th^ root of the product of *k* linear measures. This size measure is commonly used in biological- and paleo-anthropology (e.g. [45–46]) but is not yet common in strictly biological applications.

The natural log of geometric mean size (**GMS**) of skulls was calculated using the 21 major dimensions of the skull, which excludes two minor cranial measures (zygomatic height and width) due to their roughly 10-fold smaller size relative to the other dimensions, and OSA, because it is a sub-area contained within the other major dimensions of the skull. The calculation can be seen as implemented in the Excel (Microsoft Corporation) spreadsheet S1 Dataset. For eight otters for which museum records indicated original body mass of whole specimens, cranial GMS was highly correlated with body mass (r = 0.94, P < 10^−3^). Log GMS and relative log distances (log distance/log GMS) were used as size and shape measures. Log transformation of linear distances in morphometrics reduces skewness and heteroscedasticity (mean-variance autocorrelation) typical for linear distance data [47]. Log shape variables were subjected to covariance principal components decomposition to reduce the dimensionality by one, to account for the size standardization as in geometric morphometrics [44]. Principal component scores were used as shape variables for the analyses described below.

### General Morphometric Analyses

The four species were compared for size differences by analysis of variance on log GMS. Statistical analyses of form and shape were conducted with JMP and Excel. The term “form” in morphometrics is taken to mean both size and shape; “shape” is taken to mean size-standardized measures of object geometry. Therefore our analysis of form used both size and shape data as dependent variables, with feeding orientation and species nested in feeding orientation as effects. Shape analysis used only shape measures as dependent variables, with the same main effects as in the form analysis. The shape analysis also included GMS as a covariate to fit general allometry, and interactions with GMS and the main effects in case there were meaningful allometries (in nature or magnitude) specific to either feeding orientation or taxon. We applied hierarchical removal of non-significant covariates and interactions. Both two-way interactions were non-significant, so both were removed and the model was re-fit. Models were fit in Excel to check JMP output, calculate effect strengths (partial eta squared, η^2^_p_) and generate scalable multiple regression scores for visualization. One variable was noted as having a different direction of loading in the canonical space (eigenvector of **E** ^-1^ **H**) relative to that observed in least squares means and regression coefficients. In this case, we visualized multivariate regression scores (obtained by rotating centered data to the regression vector **Yβ**), instead of canonical scores.

Following multivariate analysis of variance (**MANOVA**), linear discriminant analyses (**LDA**s) with species or subspecies as the classification variable were performed in JMP to provide intuitive heuristic measures (percent of skulls correctly classified) of morphometric differentiation. Prior probabilities were set equal for all groups. Leave-one-out cross-validation (**LOOCV**) was conducted to test for overfitting of the LDAs. Overfitting can be a problem in datasets having a large suite of predictor variables relative to the number of cases. In this procedure, each case is serially excluded from LDA and predicted for class membership using the discriminant function in which it had no influence. This procedure prevents minor variations unique to specific cases from being used to generate classification success outside of the generalized group character.

### Functional Character Analyses

The kinematic (functional and ratio) variables were analyzed in univariate context due the tradition of isolated analysis in this field and a desired focus on species differences explicitly for these variables. Analysis of variance (**ANOVA**) was performed on mean MBI values to assess variation among species and among subspecies within sea otters. To test for differences in mechanical advantage at the masseter and temporalis, the in-lever/out-lever ratios were arcsine transformed [47] and an ANOVA was executed on the mechanical advantages of the temporalis and masseter to test for significant variation among species. To test for differences in occlusal surface areas (**OSA**), OSA was first regressed against condylobasal length to remove the effect of size. Residuals were then saved and used to test for significant differences among species. An ANOVA was performed with the residual OSA values as the dependent variable and species as the main effect. Post hoc contrasts (Tukey’s HSD tests) were performed on ANOVA results to discern where differences between groups lay in the univariate data space.

## Results

### Size Variation

Otter species differed in skull size (F_3,146_ = 440.9, P < 10^−18^, r^2^_adj_. = 0.90), mainly in that Asian small clawed otters were small and their size range did not overlap the other species (Tukey’s HSD, P < 0.05), though the other species, particularly the largest two species (sea otters and giant river otter), broadly overlapped in size and were not significantly different. Least square means for GMS were 3.36, 3.84, 3.60 and 3.79, respectively for Asian small clawed, sea, river and giant river otters. Within sea otters, these values were 3.83, 3.90 and 3.81, respectively, for northern, Russian and southern sea otters. Russian sea otters were larger than northern and southern sea otter subspecies (Tukey’s HSD, P < 0.05), but there was no significant difference in size between northern and southern sea otters.

### Morphometric Variation Associated with Feeding Modes (MANOVA Analyses)

**Fig 2:**
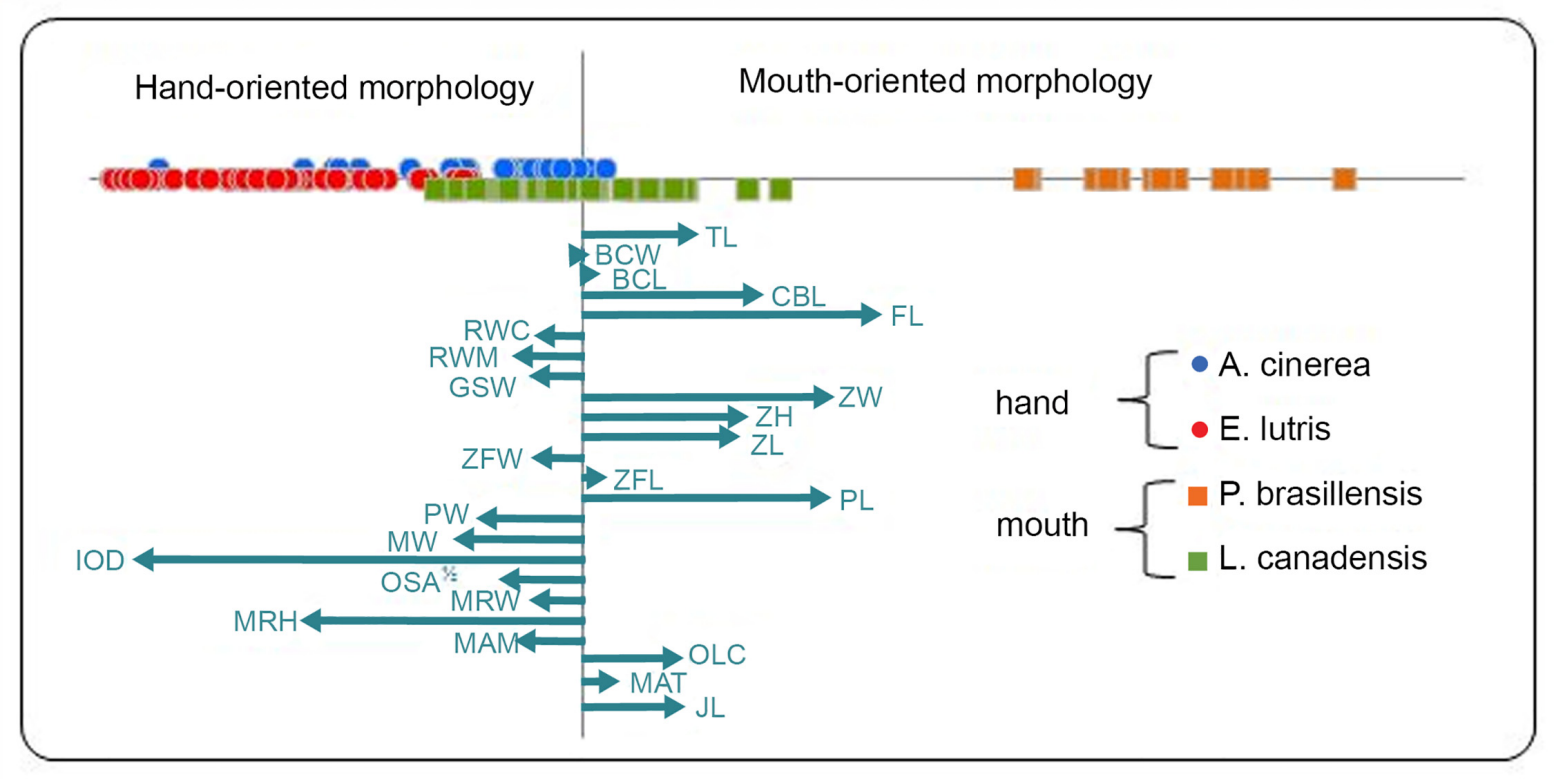
Multivariate regression ordination of skull shape based on feeding mode. Symbols reflect feeding mode for each species (circles, hand-oriented; squares, mouth-oriented). Ellipses depict standard errors for regression scores. Vectors give partial regression coefficients (trait abbreviations as in Table 1). Individuals (data points color-coded by species) and species to the right in this space have elongate skulls, especially for relative palate and jaw length, and have narrow palate width, small intra-ocular distance and shallow mandibular rami.

Patterns of allometry did not differ between foraging specialty, or species nested in foraging specialty (P ≥ 0.05), so these interactions were removed from the MANOVA. Statistical results and effect strengths for the form and shape analyses are given in Table 2. The main effect of foraging type was similar in both nature and magnitude in both models. The correlation of feeding orientation canonical scores for shape variables in the form and shape analyses was high (r = 0.93), despite the presence of strong but general allometry. Therefore, allometry did not participate strongly in creating the shape differences observed between otter foraging types. Also, there was no consistent size difference between the hand- and mouth-oriented feeders, precluding general allometry from strongly participating in the main shape effect due to feeding orientation. Therefore, we only present an ordination graphically for the shape analysis (Fig 2).

**Table 2:** MANOVA results for otter cranial form and shape.

Model effect	*Form*					*Shape*				
	F*	df_num_	df_denom_	P	ƞ_p_^2^	F*	df_num_	df_denom_	P	ƞ_p_^2^
										
Feeding orientation	103.5	24	123	< 10^−69^	0.953	68.3	23	123	< 10^−58^	0.927
Species [feeding orientation]	207.9	48	246	< 10^−172^	0.976	65.7	46	246	< 10^−113^	0.925
Size	n/a					42.3	23	123	< 10^−46^	0.888

*Exact or approximate F based on Wilks’ Λ.

Shape differentiation by foraging specialty overwhelmingly involved skull aspect ratio—hand oriented feeders had relatively short, wide shapes relative to the elongate, narrow skulls of mouth-oriented otters (Fig 2). For example, the mouth-oriented feeders had long skulls in general, with antero-posteriorly long palates, zygoma and mandibles. Hand-oriented feeders had wide palates (PW, RWM and RWC), skulls and larger interorbital distances. Additionally, the hand-oriented feeders had relatively larger mandibular rami (dorso-ventrally) and moment arm of the masseter. Hand-oriented feeders also had greater occlusal surface area (Fig 2), although this relationship was obscured in the canonical space. Since canonical spaces can become distorted by patterns in error (e.g., intraspecific shape gradients), we presented in Fig 2 the multivariate regression vectors, rather than canonical vectors. All effects in our models were very strong (0.89 < η_p_^2^ < 0.98), for all predictors in both form and shape analyses (Table 2). Discriminant analysis correctly classified over 99% of otters to their feeding specialization using both form and shape data and over 97% for form and shape in the validation procedure (S1 and S2 Tables). Classification into subspecies for each feeding specialization was over 97% for both form and shape but the validation procedure yielded lower predictive success: 76.5% for form and 77.9% for shape.

**Fig 3:**
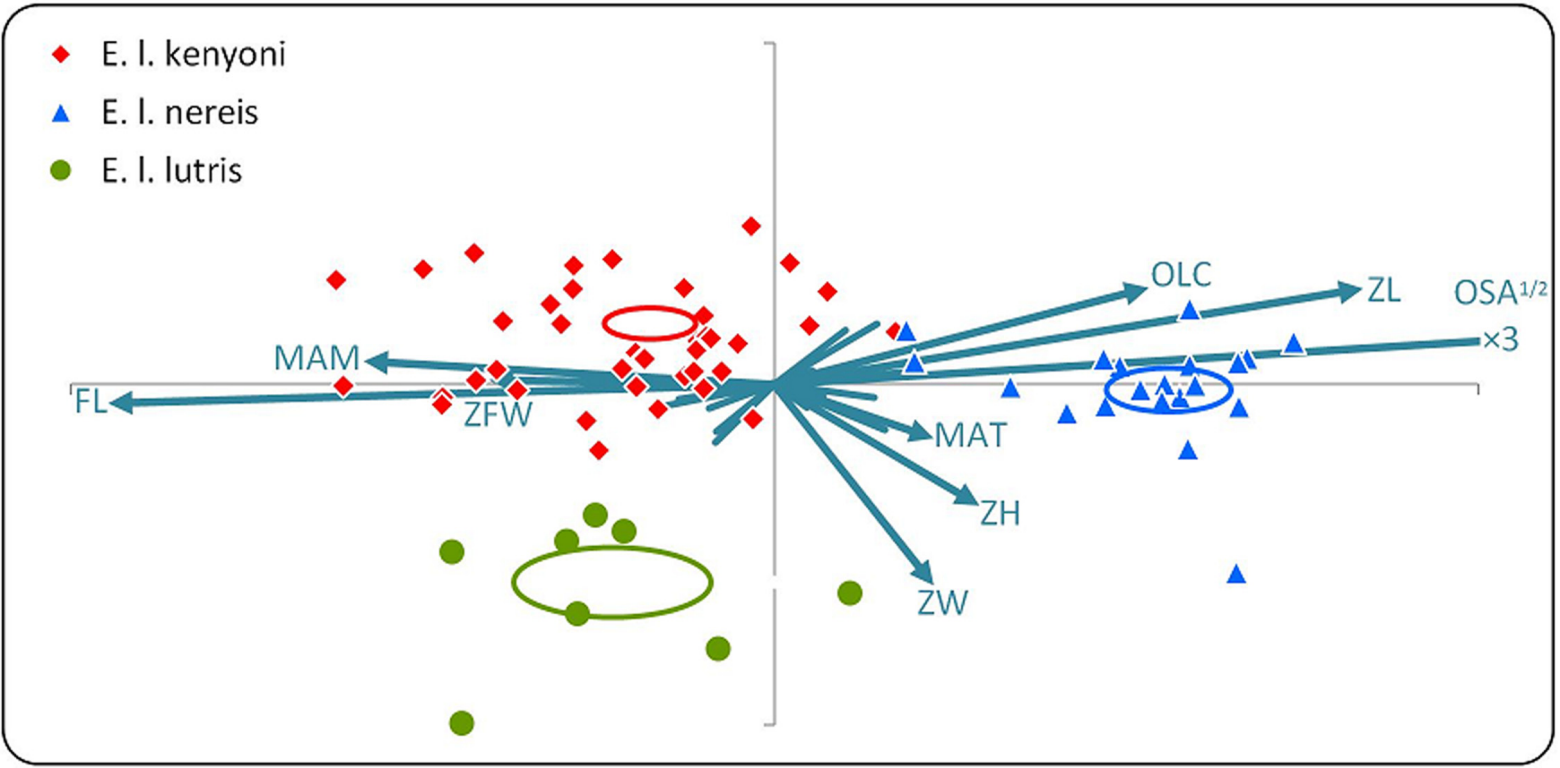
Canonical ordination of sea otter (*E*. *lutris*) skull form by subspecies. Symbols reflect feeding mode, which varies from relative piscivory (circles) to greater inclusion of invertebrates (diamonds), to durophagy (triangles). Vectors give covariance structure coefficients and only major vectors are labeled (trait abbreviations as in Table 1). The vector for OSA^½^ proceeds approximately three-fold more than shown. Ellipses depict 95% confidence intervals for subspecies centroids. The horizontal axis generally reflects the classic morphological suite of traits for durophagy: individuals to the right in this space have greatly increased occlusal surface area, long zygomas, short outlever at the level of the carnassials, short faces, a relatively short moment arm of the masseter, and relatively narrow zygomatic fossa in the center of the fossa. Plot aspect ratio (1.4:1) reflects the square root of the eigenvalue ratio for the major (horizontal) and minor (vertical) axes of skull form.

Among sea otters, southern sea otters exhibited 19.1% greater occlusal surface area than the other two subspecies, using the size-adjusted data. This effect dominated the differences observed in form and shape analyses. Shape and form analyses yielded very similar patterns (correlations between major axes: r = 0.93; for minor axes: r = 0.97), therefore only form results are presented graphically in Fig 3. Southern sea otters had longer zygoma and out-levers at the condyle. Northern and Russian sea otters possessed relatively larger faces and moment arms of the masseter, and wider zygomatic fossae.

### Mandibular Bluntness Index and Mechanical Advantage

Significant differences in the mandibular bluntness index (MBI) occurred among the four otter species (F_3,141_ = 181.9; P < 10^−47^). Post hoc tests indicated that the greatest difference among species was that sea otters possessed MBIs approximately 20% greater than the other species (Tukey’s HSD, P < 0.05). Only sea otters had MBI > 1 (least squares mean = 1.10), indicating that their mandibles were wider than long. The other species had relatively similar mean MBI, all < 1 (0.94, 0.92 and 0.91; for Asian small clawed, river, and giant river otters, respectively). MBI differentiation was also noted within sea otters, among subspecies (F_2,65_ = 3.36, P = 0.041). Post hoc tests indicated the major difference was that southern sea otters had higher MBI than northern sea otters (Tukey’s HSD, P < 0.05), and there was little difference between northern and Russian sea otters. Means for the three subspecies were 1.08, 1.08 and 1.12 for northern, Russian and southern sea otters, respectively. So, southern sea otters among the subspecies of sea otter had particularly blunt mandibles.

Significant differences in the masseteric mechanical advantage (MA_masseter_) were shown among otter species (F_3,146_ = 63.4; P < 10^−25^). Giant river otters and Asian small-clawed otters possessed the lowest MA_masseter_ and did not differ from each other (Tukey HSD, P > 0.05)—all other pairwise differences were significant in post hoc comparisons (Tukey HSD, P < 0.05). Means for the species were 0.42, 0.59, 0.53 and 0.40 for Asian small clawed, sea, river and giant river otters, respectively. Among sea otter subspecies, northern sea otters possessed a greater MA_masseter_ than southern sea otters (Tukey HSD, P < 0.05), though southern and Russian sea otters had similar means. For all subspecies, mean values were 0.61, 0.57 and 0.56 for northern, Russian and southern sea otters, respectively.

Significant differences were also detected in the mechanical advantage of the temporalis (MA_temporalis_) among species (F_3,146_ = 14.9; P < 10^−7^). The rank order of means (smallest to largest) for the otter species was giant river (0.79) > sea (0.93) > Asian small clawed (0.97) > river (1.05). All species contrasts were significant (P < 0.05) except for small clawed versus either river or sea otters (Tukey’s HSD). No significant differences were observed among sea otter subspecies (F_2,64_ = 0.82; P = 0.45).

## Discussion

Our analyses of skull morphology of four species of otters demonstrated many morphological and biomechanical differences that support divergent feeding modes of otters (i.e., mouth-oriented versus hand-oriented). Giant river otters possessed the greatest total skull length, palate length, rostrum length, and mandibular length compared to any other otter species investigated, regardless of skull size. Not surprisingly, longer skulls were correlated with longer mandibles and a bite point (at all bite locations; e.g., canines, molars) positioned farther from the temporal-mandibular joint (TMJ). Based on leverage mechanics, this should produce jaws with greater velocity. There is a mechanical tradeoff in any lever system that constrains jaws to either maximize force or velocity, but not both simultaneously [47–50]. Giant river otters are primarily piscivorous [51–52] and are mouth-oriented predators [23]. This species possessed the smallest MA_temporalis_ and MA_masseter_, which should maximize jaw velocity, an advantageous trait for capturing fast, elusive prey with the canines [52]. Mechanical advantage and gape cannot be simultaneously maximized, therefore relative increase in one may be associated with a decrease in the other [53–56].

### Interspecific Morphometric and Functional Comparisons

North American river otters primarily feed on fish, but also incorporate crustaceans (e.g., crayfish), amphibians, birds, and mollusks [57]. This diet places them intermediate between piscivorous and invertebrate specialists. Males form larger groups after the mating season concurrent with the availability of schooling fishes, such as salmon (Salmonidae), herring (Clupeidae), sandlance (Ammodytidae), and capelin (Osmeridae) [58]. These fast, schooling fishes are calorically rich and therefore may be more desirable than other prey choices. Similar to giant river otters, North American river otters possessed relatively long mandibles with a relatively low MBI, which should place the resulting force (any bite location such as canines, carnassials, back molars) farther from the TMJ, again providing the advantage of high velocity jaws to catch fast prey in open water. North American river otters possessed a higher MA_temporalis_ than any other otter species investigated, but had lower MA_masseter_. They also possessed the greatest coronoid length, an insertion of the temporalis muscle, which increases surface area for muscle attachment and correlates with a larger moment arm of temporalis. Functionally this explains the high MA_temporalis_ [5, 9,59]. The increased MA_temporalis_ functions to position force at the anterior mandible at the canines [60], which would be advantageous for a mouth-oriented predator that also may consume larger prey (e.g., salmonids).

Asian small-clawed otters, unlike giant river otters and North American river otters, are hand-oriented predators [27–28] and should not require high velocity jaws to capture prey. This expectation was supported by the morphometric analysis. Asian small-clawed otters, like North American river otters, consume a broader spectrum of prey than sea otters and giant river otters, which may be why these two species fell out relatively intermediately on of the feeding type axis, representing perhaps greater feeding generalization and making for a magnitude of variation within foraging types similar to that between foraging types (Fig 2, Table 2). The MA_temporalis_ of Asian small-clawed otter was significantly greater than the MA_masseter_. This suggests increased force at the anterior jaws near the canines. This species also possessed the greatest zygomatic fossa length. The large zygomatic fossa length should allow for a larger temporalis muscle to attach to the coronoid process of the mandible.

Sea otters are hand-oriented foragers prone to durophagy. This feeding trait is particularly pronounced in the southern sea otter subspecies. Northern sea otters that range along the Aleutian Islands and Russian sea otters also prey on epibenthic fish [28]. The main morphological traits correlated with the sea otter’s durophagy involve the extremely blunt mandible and concomitantly wide skull. This overall architecture explains the numerous differences in sea otter skull morphology compared to the other species. For example, increased greatest interorbital distance, rostral width at the molars, braincase width, palate width, and greater skull width are all correlates of the extremely blunt and wide cranium and mandible exhibited by sea otters. The corresponding differences in jaw adductors likely explain the increased zygomatic length. The MA_masseter_ of sea otters was significantly greater than any other otter species investigated. Increased MA_masseter_ functions to increase force at the caudal jaw near the molars and should be considered an adaptation for durophagy. A greater moment arm of the masseter also generates more control over mastication [61]. The height of the mandibular ramus and depth of the mandibular fossa should also relate to increased bite force. The length and depth of the masseteric fossa was enlarged in sea otters, providing additional surface area for the masseter to insert as well as likely accommodating a masseter with a relatively larger physiological cross-sectional area (PCSA). However, PCSA could not be measured directly in this study because the work was performed on curated skulls. Our morphometric analysis of sea otters demonstrated that they exhibited taller and wider mandibular rami, and shorter, blunter skulls than other otters. Similar cranial characteristics have been demonstrated to increase bite force at the carnassials in other carnivorans [5, 9, 53, 62]. An increase in the vertical height of the mandibular ramus, results in an increase in vertical orientation of the masseter [63]. This functions to increase the moment arms of the masseter and medial pterygoids [63]. One possible downside to this arrangement is that a greater mandibular ramus height and a more rostral position of the masseter could result in a reduced gape, unless the muscle architecture is modified. However this could be accomplished by increasing muscle fiber length [64]. Sea otters have been observed to display wide gapes (approximately 61 to 66°) in comparison to other extant mammals [65]. In addition to shorter facial lengths (which also positions the masseter muscle more anteriorly), sea otter mandibles have relatively tall and vertically-oriented rami combined with a long zygomatic arch for masseter attachment. A long zygomatic length allows muscles to attach more anteriorly, thus improving bite force while allowing a larger gape.

In general, mammals with a large gape angle require muscles that stretch, which impacts mechanical advantage [53–55, 60]. The underlying basis for this is the optimal range for sarcomere length and the classic muscle tension curve. Mammalian sarcomeres reach peak tension at approximately 2.4–2.8 μm [66–68]. Within this optimal range, the maximum number of cross bridges can form during muscle contraction and the highest muscle tension is produced. When sarcomeres are completely shortened, the thick filaments are pressed against the Z-lines and the myosin heads cannot pivot or produce as much tension. Alternatively, if sarcomere lengths are greater than the optimal range, tension is reduced by the reduction of the overlap zone and the number of cross-bridge interactions between actin and myosin [69–71]. The latter situation occurs at larger gapes unless certain other adaptations are in place. The architecture of sea otter jaw adducting muscles may be modified allowing for greater stretch [72]. The stretch factor is the ratio of L/l, where L is the length of the muscle at maximum gape and l is the length of the muscle when the mouth is closed [54]. The stretch factor can be varied by changing the origin-insertion ratio (e.g., the origin may migrate anteriorly) and the lengthening of individual fibers (by reducing pennation), which allows for greater gape [54].

Longer muscle fibers can increase maximum muscle excursion (i.e., the distance a muscle fiber can shorten) [73]. For jaw adducting muscles, this likely translates to wider maximum gapes [73]. The masseter of common marmosets (*Callithrix jacchus*) and pygmy marmosets (*Cebuella pygmaea*), both tree gouging primates, displayed longer fiber lengths than non-gouging tree primates, which was correlated with greater gape [73]. Similar results (long fiber lengths and large gape) have been reported in pigs [54, 74] and mice [75]. Craniodental morphometric and kinematic data indicate that sea otters exhibit a wide gape [65] and we predict that sea otter adductor muscle architecture and physiology are modified to allow for a wide gape while maintaining a high bite force.

Sea otters possess large occlusal surface areas (OSA) of the postcanine teeth. The broad flat occlusal plane provides a large surface to crush prey [76]. The broad postcanine teeth of sea otters are consistent with other mustelids that crush their prey [14]. When the effect of size was eliminated, relative OSA of sea otters was the largest of all otters investigated, even larger than giant river otters [51, 62]. In more piscivorous otter species (e.g., giant river otters), the postcanine teeth possess sharp cusps ideal for slicing prey [19, 76]. However, sea otters are the only extant otter that possesses bunodont dentition [19].

Unlike all other otter species that had long and narrower rostrums, sea otters also possessed a greater height of the rostrum. While such increases may serve for increased area for muscle attachment [77], the increased bite force generated by shorter skull lengths [62] may produce rostral bending, or torsion, that would be resisted by a taller, wider rostrum [39, 78–80]. The more anteriorly positioned zygomatic arch and shortening of the rostrum are also characteristic of animals that consume hard prey [62]. Shorter, blunter jaws (MBI > 1.0) place the resulting force closer (any bite location such as canines, carnassials, back molars) to the TMJ, which provides the advantage of increased MA_masseter_ and thus contributes to a greater bite force performance. Shorter and wider skulls also bring the canines closer to the fulcrum, which increases the MA_masseter_ even at the tip of the jaw, resulting in increased bite force at this position as well. A similar situation (shorter and wider skulls) has been reported for phyllostomid bats in which direct bite force at the jaw tips was measured [62]. Therefore, the extreme blunt and short skulls, and postcanine morphology of sea otters show numerous advantageous traits for specializing on hard, benthic prey. These specializations for durophagy would be inconsistent with a mouth-oriented prey capture strategy. All craniodental morphologies and jaw biomechanics demonstrate that sea otter skulls are built to maximize bite performance and crush prey.

### Intraspecific Morphometric and Functional Comparison of Sea Otter Subspecies

Variation was also observed in the cranial morphology among sea otter subspecies. Russian sea otters possessed greater palatal and zygomatic fossa lengths compared to the other two subspecies. Southern and northern sea otters possessed greater zygomatic lengths than Russian sea otters, which increase the surface area for masseter attachment. As mentioned above, an increase in zygomatic length can increases MA_masseter_ [54]. Northern sea otters displayed the greatest MA_masseter_, followed by southern and Russian sea otters. Northern sea otters also exhibited greater braincase and zygomatic fossa width than southern and Russian sea otters. A large zygomatic fossa width allows a large temporalis muscle cross-sectional area and mass to connect with the coronoid process. This should function to increase the in-force component of the jaw lever and the MA_temporalis_ [50, 81–83]. The greater MA_masseter_ and MA_temporalis_ should correspond to greater estimated bite forces but also negatively influence jaw velocity. Southern sea otters and northern sea otters consume a greater quantity of hard, benthic prey [32, 84–91], which require a greater bite force than is required for fish. Russian and northern sea otters inhabiting parts of southwest Alaska including Alaska Peninsula, Aleutian Islands, and Amchitka Island include epibenthic fish such as flatfish (Pleuronectids) in their diet [28, 85, 92]. However, populations in Southwest Alaska still incorporate more benthic invertebrates than fish into their diets [85, 92]. Russian sea otters, on the other hand, incorporate a larger proportion of fish in their diets [28], which may correspond with smaller MA_masseter_ and estimated bite forces compared to southern and northern sea otters.

## Conclusions

The morphometric analysis and kinematic deductions herein indicate strong divergent skull and dental patterns concomitant with two behavioral trophic specializations, mouth- versus hand-oriented foraging, in otters. The combined morphological differentiation and alternative foraging strategies provide access to prey that on one extreme are elusive, open-water organisms that do not require crushing (e.g., fish), and at another extreme are relatively non-elusive, benthic hard-bodied organisms (e.g., crustaceans, molluscs). North American river otters and giant river otters are mouth-oriented predators that possess long mandibles, rostrums, and long and gracile pterygoid hamuli. Longer, narrower skulls and long mandibles position the resulting bite force farther from the temporomandibular joint, providing jaws with greater velocity at the expense of bite force capability. High velocity jaws are an important adaptation for mouth-oriented species that prey upon fast moving prey, such as fish. Sea otters and Asian small-clawed otters are hand-oriented predators with further modified dentition (e.g., wide, flat occlusal surfaces) and shorter skulls and mandibles than North American river and giant river otters. Shorter jaws position the resulting bite force (bite location, canines, carnassials, molars) closer to the TMJ, providing a more forceful bite at the expense of velocity.

The morphological and functional clarity of the differences among four species employing the alternative foraging modes begs the question of whether the trait differences evolved only once or more times in the otter lineage. Well-resolved otter phylogenies by Koepfli and colleagues [93, 94] are presented, trimmed to the four species used in the present study, in S1 Text. The phylogenies evince long branches for all species with crowded but fully-resolved insertions near the base. Despite the near polytomy, the resolved basal branches with both hand-oriented species on a single derived branch suggest a single, deeply-rooted evolutionary transition in foraging mode and concomitant shift in morphology. This might lend one to believe that foraging mode and morphology is a vestige of ancient selection. So it is fortuitous that our analysis also included the subspecific component.

It is particularly compelling that the intraspecific variation we examined was similarly pronounced, with no overlap among subspecies in the canonical trait space (Fig 3) and composed of similar elements of anatomical variation, as the interspecific differentiation across the functional continuum of foraging on elusive, open-water prey versus relatively less mobile, hard-bodied prey (Fig 2). Since variation at the sub-specific level by definition reflects more recent evolution than the species phylogeny, it is clear that trophic differentiation may be either deeply- or shallowly-rooted and is at least partly replicable (*sensu* reference [95]). Reference to other otter species foraging modes, though in the absence of detailed morphological analysis, also suggests this evolutionary lability as at least two bifurcations in the larger lineage of otters would be required to produce the foraging characters we noted in the Introduction for other species of otter. Thus, the hand- and mouth-oriented behavioral and morphological suite will be interesting to examine in more species of otters, or broadened taxonomically and functionally to encompass more traits and ecological axes. The differentiation we observed within and among otter species in the present study is testament to the power of both natural selection and the functional approach to studying organismic variation.

## Supporting Information

S1 Dataset Cranio-dental morphology datasheet. (XLS)

S1 Text Phylogenetic trees and variance-covariance matrices for four otter species. Phylogenetic trees were extracted from the larger phylogenetic analyses by Koepfli, Wayne and colleagues [92, 93], as depicted below. Mouth-oriented feeders have been considered basal (ancestral) [21, 24], which is also supported in the trimmed phylogenies. Although the phylogenies have a shape verging on polytomy, the basal insertions are fully resolved [93]. Therefore, mapping the feeding-type trait, indicated by different colors below, indicates a single evolutionary event. Hand-oriented feeding, and the concomittant morphology, would be considered a single derived state as indicated by the red branches below.
(DOC)

S1 Table Confusion matrices reporting prediction success of otters into four species using linear discriminant analysis (LDA) of skull form (left) and shape (right). Species acronyms are (Aoci, *Aonyx cinerea*; Enlu, *Enhydra lutris*; Loca, *Lontra canadensis*; Ptbr, *Pteronura brasiliensis*). Upper tables give results for the analysis including all data. Lower tables give summaries of leave-one-out cross-validation runs wherein each datum was predicted from an LDA in which it was excluded from calculating discriminants. Numbers in red boldfaced font indicate misclassifications.
(DOCX)

S2 Table Confusion matrices reporting prediction of otters into three sea otter (*Enhydra lutris*) subspecies using linear discriminant analysis (LDA) of skull form (left) and shape (right). Subspecies acronyms are (ken, *E*. *l*. *kenyoni;* lut, *E*. *l*. *lutris*; ner, *E*. *l*. *nereis*). Upper tables give results for the analysis including all data. Lower tables give summaries of leave-one-out cross-validation runs wherein each datum was predicted from an LDA in which it was excluded from calculating discriminants. Numbers in red boldfaced font indicate misclassifications.
(DOCX)
