# Supplementary material for: Divergent Skull Morphology Supports Two Trophic Specializations in Otters (Lutrinae)
Source: PLoS One. 2015 Dec 9;10(12):e0143236. doi: 10.1371/journal.pone.0143236 (PMC4674116; doi:10.1371/journal.pone.0143236)
Supplement: S1 Text — Phylogenetic trees were extracted from the larger phylogenetic analyses by Koepfli, Wayne and colleagues [92, 93], as depicted below. Mouth-oriented feeders have been considered basal (ancestral) [21, 24], which is also supported in the trimmed phylogenies. Although the phylogenies have a shape verging on polytomy, the basal insertions are fully resolved [93]. Therefore, mapping the feeding-type trait, indicated by different colors below, indicates a single evolutionary event. Hand-oriented feeding, and the concomittant morphology, would be considered a single derived state as indicated by the red branches below. (DOC) [file pone.0143236.s002.doc]

**S1 text. Phylogenetic trees and variance-covariance matrices for four otter species**

Phylogenetic trees were extracted from the larger phylogenetic analyses by Koepfli, Wayne and colleagues [93, 94], as depicted below. Mouth-oriented feeders have been considered basal (ancestral) [21, 24], which is also supported in the trimmed phylogenies. Although the phylogenies have a shape verging on polytomy, the basal insertions are fully resolved [94]. Therefore, mapping the feeding-type trait, indicated by different colors below, indicates a single evolutionary event. Hand-oriented feeding, and the concomittant morphology, would be considered a single derived state as indicated by the red branches below.

Koepfli et al. (2008)

Acin

Elut

Pbra

Lcan

1

1

17

10

15

12

Koepfli & Wayne (2002)

mitochondrial Cyt C

Elut

Lcan

Acin

Pbra

205

171

225

131

40

28

Koepfli et al. (2008)

22 various genes

However, if one looks at the broader clade for the Lutrinae, though lacking morphological data as presented in this paper for the four focal taxa, there appears to be at least two evolutionary events of foraging mode transition. In addition, our analysis of subspecies variation in sea otters indicates another transition in the generalized behavioral/morphological trait suite at the tip of the sea otter branch of the phylogeny.

Collectively then, feeding-type and morphology in otters appears to be both evolutionarily dynamic and replicable in terms of the morphological component of diversification.

It is worth noting that our study involved only four taxa, two species nested in each of two feeding types. The analysis used in the main text was a nested GLM and so is basically a single degree of freedom contrast for the foraging mode effect as is appropriate to the phylogenetic structure at hand. That said, it is interesting that the morphological associations with foraging mode should have persisted from a very old shared node (ancestor) to present, especially since the species nested in foraging mode term is so strong, indicating plenty of morphological evolution but not erasure of the generalized foraging morphologies. This implies a fair amount of stabilizing selection maintaining the foraging morphologies in these clades.
