# Supplementary material for: Divergent Skull Morphology Supports Two Trophic Specializations in Otters (Lutrinae)
Source: PLoS One. 2015 Dec 9;10(12):e0143236. doi: 10.1371/journal.pone.0143236 (PMC4674116; doi:10.1371/journal.pone.0143236)
Supplement: S1 Table — Species acronyms are (Aoci, Aonyx cinerea; Enlu, Enhydra lutris; Loca, Lontra canadensis; Ptbr, Pteronura brasiliensis). Upper tables give results for the analysis including all data. Lower tables give summaries of leave-one-out cross-validation runs wherein each datum was predicted from an LDA in which it was excluded from calculating discriminants. Numbers in red boldfaced font indicate misclassifications. (DOCX) [file pone.0143236.s003.docx]

|  |  | **FORM** | | | | |  |  |  | **SHAPE** | | | | |
| --- | --- | --- | --- | --- | --- | --- | --- | --- | --- | --- | --- | --- | --- | --- |
|  |  |  |  |  |  |  |  |  |  |  |  |  |  |  |
|  |  |  | Predicted | | | |  |  |  |  | Predicted | | | |
|  |  |  | Aoci | Enlu | Loca | Ptbr |  |  |  |  | Aoci | Enlu | Loca | Ptbr |
| All-inclusive | Actual | Aoci | 23 | 0 | 0 | 0 |  | All-inclusive | Actual | Aoci | 22 | 0 | 0 | 0 |
|  |  | Enlu | 0 | 68 | 0 | 0 |  |  |  | Enlu | **1** | 68 | 0 | 0 |
|  |  | Loca | 0 | 0 | 42 | 0 |  |  |  | Loca | 0 | 0 | 42 | 0 |
|  |  | Ptbr | 0 | 0 | 0 | 17 |  |  |  | Ptbr | 0 | 0 | 0 | 17 |
|  |  |  |  |  |  |  |  |  |  |  |  |  |  |  |
|  |  |  | Predicted | | | |  |  |  |  | Predicted | | | |
|  |  |  | Aoci | Enlu | Loca | Ptbr |  |  |  |  | Aoci | Enlu | Loca | Ptbr |
| LOOCV | Actual | Aoci | 23 | 0 | 0 | 0 |  | LOOCV | Actual | Aoci | 20 | **1** | 0 | 0 |
|  |  | Enlu | 0 | 68 | 0 | 0 |  |  |  | Enlu | **3** | 67 | 0 | 0 |
|  |  | Loca | 0 | 0 | 42 | 0 |  |  |  | Loca | 0 | 0 | 42 | 0 |
|  |  | Ptbr | 0 | 0 | 0 | 17 |  |  |  | Ptbr | 0 | 0 | 0 | 17 |

**S1 Table.** **Confusion matrices reporting prediction success of otters into four species using linear discriminant analysis (LDA) of skull form (left) and shape (right).** Species acronyms are (Aoci, *Aonyx cinerea*; Enlu, *Enhydra lutris*; Loca, *Lontra canadensis*; Ptbr, *Pteronura brasiliensis* ). Upper tables give results for the analysis including all data. Lower tables give summaries of leave-one-out cross-validation runs wherein each datum was predicted from an LDA in which it was excluded from calculating discriminants. Numbers in red boldfaced font indicate misclassifications.
