# Supplementary material for: Divergent Skull Morphology Supports Two Trophic Specializations in Otters (Lutrinae)
Source: PLoS One. 2015 Dec 9;10(12):e0143236. doi: 10.1371/journal.pone.0143236 (PMC4674116; doi:10.1371/journal.pone.0143236)
Supplement: S2 Table — Subspecies acronyms are (ken, E. l. kenyoni; lut, E. l. lutris; ner, E. l. nereis). Upper tables give results for the analysis including all data. Lower tables give summaries of leave-one-out cross-validation runs wherein each datum was predicted from an LDA in which it was excluded from calculating discriminants. Numbers in red boldfaced font indicate misclassifications. (DOCX) [file pone.0143236.s004.docx]

|  |  | **FORM** | | | |  |  |  |  | **SHAPE** | | | |
| --- | --- | --- | --- | --- | --- | --- | --- | --- | --- | --- | --- | --- | --- |
|  |  |  |  |  |  |  |  |  |  |  |  |  |  |
|  |  |  | Predicted | | |  |  |  |  |  | Predicted | | |
| All-inclusive | Actual |  | ken | lut | ner |  |  | All-inclusive | Actual |  | ken | lut | ner |
|  |  | ken | 40 | 0 | 0 |  |  |  |  | ken | 39 | 0 | **1** |
|  |  | lut | 0 | 8 | 0 |  |  |  |  | lut | 0 | 8 | 0 |
|  |  | ner | 0 | 0 | 20 |  |  |  |  | ner | **1** | 0 | 19 |
|  |  |  |  |  |  |  |  |  |  |  |  |  |  |
|  |  |  |  |  |  |  |  |  |  |  |  |  |  |
|  |  |  | Predicted | | |  |  |  |  |  | Predicted | | |
| LOOCV | Actual |  | ken | lut | ner |  |  | LOOCV | Actual |  | ken | lut | ner |
|  |  | ken | 32 | **4** | **4** |  |  |  |  | ken | 32 | **5** | **3** |
|  |  | lut | **3** | 4 | **1** |  |  |  |  | lut | **3** | 5 | 0 |
|  |  | ner | **3** | **1** | 16 |  |  |  |  | ner | **3** | **1** | 16 |
|  |  |  |  |  |  |  |  |  |  |  |  |  |  |

**S2 Table.** **Confusion matrices reporting prediction of otters into three sea otter (*Enhydra lutris*) subspecies using linear discriminant analysis (LDA) of skull form (left) and shape (right).** Subspecies acronyms are (ken, *E. l. kenyoni;* lut, *E. l. lutris*; ner, *E. l. nereis*). Upper tables give results for the analysis including all data. Lower tables give summaries of leave-one-out cross-validation runs wherein each datum was predicted from an LDA in which it was excluded from calculating discriminants. Numbers in red boldfaced font indicate misclassifications.
